# Integrating Generative AI Into Patient-Centered Clinical Decision Support: Viewpoint on Research and Practice Considerations

**DOI:** 10.2196/81628

**Published:** 2026-04-01

**Authors:** Prashila Dullabh, Courtney Zott, Nicole Gauthreaux, Caroline Peterson, Abigail Aronoff, Kistein Monkhouse, Dean F Sittig

**Affiliations:** 1 Health Sciences Department, NORC at the University of Chicago, 1828 L Street NW, 9th Floor, Washington, DC, 20036, United States, 1 202-280-9294; 2 Patient Orator Inc, New York, NY, United States; 3 Informatics Review LLC, Lake Oswego, OR, United States

**Keywords:** generative artificial intelligence, clinical decision support systems, patient-centered care, patient involvement, artificial intelligence, AI, quality of health care

## Abstract

There is growing interest in understanding how generative artificial intelligence (GenAI) can support patients and caregivers in making informed health care decisions, known as patient-centered clinical decision support (PC CDS). In this viewpoint, we present example applications for GenAI-supported PC CDS for patients, caregivers, clinicians, and patient-clinician interactions and examine the opportunities, challenges, and potential solutions associated with these applications. We conducted a targeted document review of our work in the Agency for Healthcare Research and Quality’s Clinical Decision Support Innovation Collaborative focusing on GenAI-enabled PC CDS, supplemented by snowball sampling and targeted searches to identify additional applications. Findings were refined and validated through solicited feedback from a 20-member multidisciplinary expert panel. Through our work, we highlight six critical needs that must be addressed to fully realize GenAI’s potential in PC CDS: (1) engage and ensure representation of patients and caregivers in design and development; (2) build the science of effective PC CDS implementation to support patient engagement; (3) develop risk-based policies for when to use GenAI; (4) establish independent testing and vetting criteria; (5) periodically reassess to identify and address algorithmic drift and verify performance; and (6) establish policies to promote transparency and patient consent in the use of GenAI. Understanding the applications and their potential implications for health care quality is essential to further the beneficial, ethical, and safe development of GenAI-supported PC CDS.

## Introduction

The clinical landscape is experiencing rapid growth in artificial intelligence (AI), especially generative AI (GenAI) systems that learn the underlying structure of their training data to create new content based on those learned patterns, such as new text, images, and data prompts [1]. This technology has sparked interest in its potential to integrate new data and analytics into patient-centered clinical decision support (PC CDS). PC CDS encompasses decision-making tools that significantly incorporate patient-centered factors across 4 key dimensions: knowledge (evidence based on comparative effectiveness research and research findings on patient-centered outcomes), informed by data that are directly generated from patients (ie, patient-generated health data [PGHD], patient-reported outcomes [PROs], and/or preferences), delivery (engaging patients and caregivers across settings through patient portals, apps, and other digital tools), and use (facilitating shared decision-making [SDM], a process where patients, caregivers, and care teams share and discuss health information and patients’ values and preferences to reach mutually acceptable health-related decisions) [2]. Studies have suggested that GenAI has the potential to impact health care delivery, facilitate more personalized care, influence patient outcomes, and reshape the clinician-patient and clinician-caregiver relationship [3-5]. At the same time, researchers have identified barriers to realizing GenAI’s potential, including limited GenAI implementation frameworks that consider the full sociotechnical environment [6], variability in requirements for reporting and validating GenAI [7], and mixed perceptions about the utility and safety of the technology among clinicians and patients [8]. Additionally, while many articles have described GenAI’s short-term impact on clinicians and health care systems, such as relieving clinician workload and improving operational efficiency, to our knowledge, relatively few focus on the decision-making tools that support patient-centered care or on tools where the patient is the user [9-11]. Because these tools directly engage patients in understanding health information, managing their conditions, and participating in care decisions, they raise distinct expectations around health literacy, such as supporting patients’ ability to obtain, process, and understand health information [12]; self-management [13], such as strengthening patients’ problem-solving, decision-making, and action-planning skills; and SDM, such as enhancing patients’ knowledge, awareness, and ability to cope and engage in collaborative decisions with their care teams [1,14]. There is a need to understand the various ways and intricacies of leveraging GenAI to engage patients and caregivers in their care and how these tools can enhance patient-clinician communications and support the delivery of evidence-based care.

This viewpoint summarizes the novel applications of GenAI in PC CDS through illustrative use cases that can provide a better understanding of its potential among patients, caregivers, clinicians, and PC CDS developers. On the basis of these illustrative use cases, we identified common themes related to the benefits and challenges of GenAI-supported PC CDS tools on health care quality and delivery, with the purpose of acknowledging their advantages while mitigating their potential risks. Drawing from the challenges, we discuss ways to advance trustworthy GenAI-based PC CDS to chart a path forward for research and practice.

## Targeted Review Approach

We used 3 methods to identify the use cases.

First, we conducted an in-depth review of 4 reports from the Agency for Healthcare Research and Quality’s Clinical Decision Support Innovation Collaborative that addressed AI applications, including a landscape assessment on using AI to scale PC CDS [15], a report on patient and caregiver perspectives on GenAI in PC CDS [16], and 2 assessments of AI-supported PC CDS tools [17,18].

Second, we used a snowball sampling approach to identify additional relevant literature from the reference lists and conducted targeted searches in the PubMed and Google Scholar databases. In total, we identified 53 peer-reviewed sources. From these sources, we abstracted the type of AI technology used, the specific use cases, and the intended end users of these systems.

Third, we solicited feedback from a 20-member Steering Committee including patients, clinicians, informaticians, researchers, electronic health record (EHR) developers, payers, and policymakers. After the research team (PD, CZ, NG, and AA) completed the targeted review analysis and developed a draft use case exhibit, the lead author presented the preliminary findings to the Steering Committee and invited feedback on additional GenAI-supported PC CDS use cases to consider, the categorization of use cases, the validation of the identified benefits and considerations, and recommendations regarding critical needs for the effective use of GenAI-supported PC CDS. Meeting minutes documented key points of agreement, which were subsequently incorporated by the research team.

## Use Cases for GenAI-Supported PC CDS

**Figure 1.**
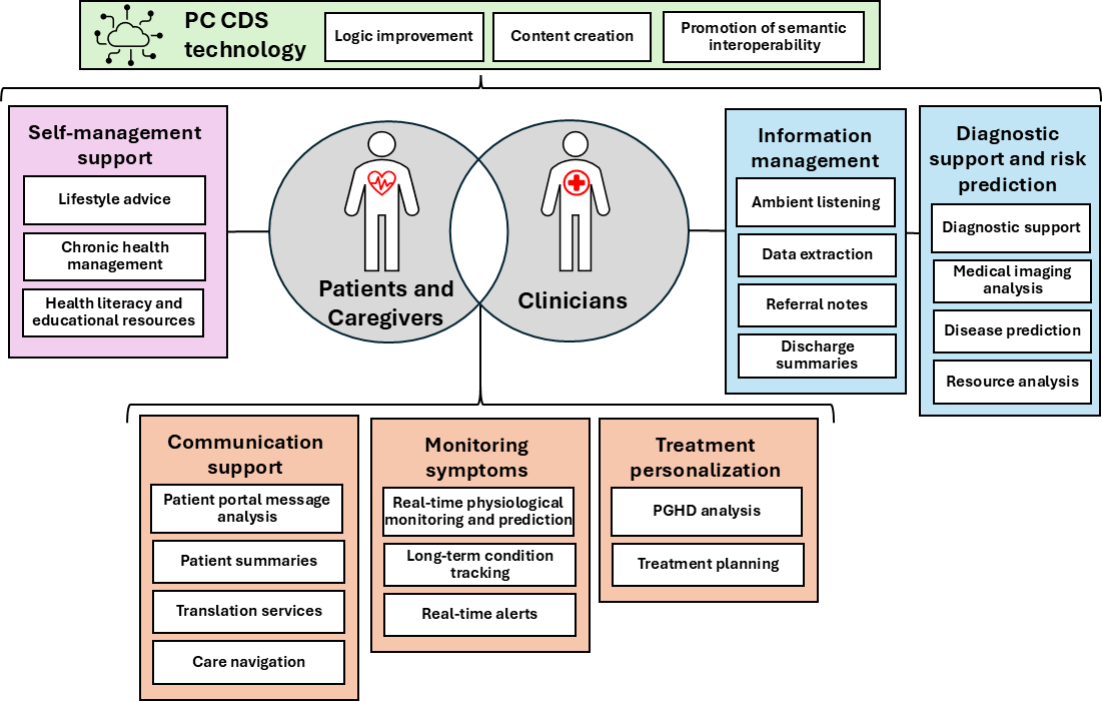
Use cases of generative artificial intelligence in patient-centered clinical decision support (PC CDS) technology. PGHD: patient-generated health data.

Figure 1 depicts use cases for GenAI-supported PC CDS that operate across categories based on their primary users and functions. Before reaching end users, GenAI can streamline the technical development of PC CDS for developers and implementers by optimizing knowledge artifacts and reducing development time and cost [15,19]. For end users, patient- and caregiver-focused tools emphasize health and well-being, chronic condition self-management, and educational resources. Tools supporting patient-clinician interactions facilitate communication through conversational agents, symptom monitoring systems, and treatment personalization for patients, which are discussed through SDM with their clinician. Clinician-focused applications assist with information management, particularly for analyzing large volumes of PGHD, while also providing diagnostic support and risk prediction. The potential impact and clinical significance of these use cases will vary across specialties, care settings, and patient populations, and the figure is not intended to imply a hierarchy of importance among them.

## Salient Benefits and Considerations on the Use of GenAI in PC CDS

### Overview

The use cases for GenAI-supported PC CDS illuminate several crosscutting themes that highlight the benefits and challenges of implementing these systems in health care settings. The themes do not apply uniformly across all use cases but rather vary in relevance depending on factors such as the clinical context, the decisions being made, and the patient-clinician interactions involved. Some themes also naturally intersect. For example, challenges such as hallucinations and misinformation cut across multiple applications and mediate a range of GenAI’s benefits. In the following discussion, the examples specify the applicable use cases and raise challenges where they are highly relevant, illustrating how these themes can manifest across different applications.

### Personalization and Precision

GenAI can support the delivery of personalized care that is responsive to a patient’s unique health profile. GenAI is equipped to rapidly process extensive amounts of patient-centered health information from a range of sources, such as patients’ EHRs, genomic data, medical devices, wearable technologies, and questionnaires assessing patients’ preferences and goals [20]. It can then leverage complex algorithms to generate personalized treatment advice and recommendations, facilitating SDM [21-25]. For example, an AI-powered remote monitoring system for diabetes can collect and analyze patients’ blood glucose levels, exercise levels, and eating habits to generate personalized meal plans and exercise routines for patients [26]. One study developed an AI-assisted Internet of Things wearable smartwatch prototype for older people to proactively detect and manage frailty through the collection of their physical activity data [27].

However, one of the key limitations of using GenAI to provide individualized health recommendations is its inconsistent reasoning capabilities and clinical judgment, particularly when it must synthesize information across long conversations, complex clinical scenarios, or high-volume data sources such as wearables. A systematic review found several issues with large language models (LLMs) such as fragile reasoning performance, diagnostic inaccuracies, and overly cautious clinical judgments across a range of use cases including treatment personalization, monitoring systems, communication support, and diagnostic support and risk prediction [28]. Others have raised concerns about personalized AI’s potential to limit exposure to critical information, fostering “echo chambers” that reinforce users’ specific beliefs [29].

### Patient Engagement and Empowerment

Emerging use cases for GenAI center on its potential to empower patients through self-management and communication support. GenAI can make health information more understandable and equip patients with the information needed to take a more active role in their care. For example, health systems can use GenAI-powered chatbots or digital health assistants as patient-facing self-management resources and/or as communication support tools for clinicians to provide health information, answer questions, help assess symptoms, share medication reminders, and facilitate appointment scheduling [30,31].

GenAI has the potential to support SDM between patients and clinicians by providing data or educational resources [32]. One study explored perspectives on the use of GenAI in SDM for knee replacement surgery, finding that patients viewed GenAI as another source of information that could enhance their understanding of their risk profile, empowering them to make decisions based on their values and preferences [10]. Despite its potential, patients with less experience using technology or navigating health information from electronic sources (ie, limited digital health literacy) may find using these tools challenging, leading to a sense of disempowerment [16]. With GenAI in particular, emerging evidence suggests that patients may mistake the confident tone of model outputs for factual accuracy, increasing the likelihood that they follow guidance that is incomplete or inaccurate [33-35].

### Quality of Care and Safety

GenAI shows promise in improving patient safety and quality of care. It can facilitate real-time physiological monitoring and prediction of patients’ conditions and send recommendations to patients or alerts to clinicians to support care coordination [36]. An ongoing study of a GenAI-supported PC CDS monitoring system for patients with asthma uses voice biomarker technology to recognize variations in the patient’s recorded voice and sends immediate alerts to the patient for treatment intervention. The digital tool calculates a Respiratory Symptoms Risk Score and allows for remote care coordination, as necessary [37]. GenAI can also enhance diagnostic accuracy and risk prediction based on patient health histories [38], unstructured clinical notes [39,40], PGHD [41], or medical imaging analysis [42]. In some cases, this can inform allocation of resources and treatments based on individual- and system-level data. Mercy Healthcare System integrated the Chen Chemotherapy Model into their EHR to predict hospitalization risk from chemotherapy side effects in adult patients without leukemia. The texting platform prompts patients to report and rate their symptoms, then sends this PRO data to clinicians for review [43]. This allows clinicians to proactively manage symptoms before hospitalization becomes necessary. Furthermore, GenAI can summarize disparate information for clinicians to allow them more time for direct patient interactions, which can improve care quality [44]. With ambient listening technology, GenAI-supported tools convert recorded patient-clinician conversations into structured visit summary notes. These tools reduce the time clinicians spend on documentation during visits, enabling them to focus more attention on direct patient communication and interaction [45].

At the same time, overreliance on GenAI can pose risks to patient safety. One study found that GenAI models exhibited larger cognitive biases for medical decisions when compared with practicing clinicians, illustrating how LLMs could lead to diagnostic or treatment errors in complex medical cases [46]. Another study found that more than half of a sample of primary care physicians reviewing AI-generated patient portal messages containing errors did not identify and remedy all the errors and 35% to 45% of physicians submitted erroneous messages entirely unedited, indicating the need for additional guardrails beyond physician oversight [47]. Additionally, liability questions arise when GenAI is involved in decision-making, especially for “black box” algorithms where the internal decision-making process is hidden, meaning users can only see the input data they provide and the resulting output without understanding the reasoning process for medical predictions or decisions [48]. This makes it more difficult to determine who is accountable if there are adverse outcomes or whether the health system should take responsibility if they do not properly implement, maintain, or train users on the GenAI-supported system [5,49].

### Access to Care

GenAI-supported PC CDS has the potential to improve access to medical care. It can support translation service use cases by using speech recognition and generating speech output in any language, thus overcoming language barriers between patients and their clinicians [50]. It can also be used for self-management support use cases in settings where there is a shortage of health care professionals, such as in rural areas or when more immediate access to clinical expertise is needed (regardless of geographic setting) to assist patients with nonurgent medical conditions. For example, Spänig et al [51] demonstrated that an autonomous medical AI interface designed to identify patients at risk for type 2 diabetes mellitus could function as an initial point of guidance in settings with limited clinician availability by offering preliminary risk assessment and directing patients toward appropriate follow-up care.

Despite this potential, GenAI systems may produce uneven health outcomes if not carefully implemented. Models trained on limited or skewed datasets could lead to less accurate self-management recommendations or physiological monitoring predictions for different patient populations [52]. Biased GenAI outputs can result from a range of common data quality issues in training models, such as limited demographic information [53,54], inaccurate or missing data [55], and unrepresentative data that do not include adequate information on specific patient subpopulations [56,57]. While bias can be mitigated by preprocessing data used to train AI models with retrieval-augmented generation (coupling models with an external and reliable database or knowledge base) [58], selecting models that prioritize transparency, and postprocessing the model output to correct for bias, these approaches require developer assessments of what constitutes fairness [59].

Beyond its potential for bias and error, a wide range of factors limit GenAI’s integration into patients’ lives, which can influence access to care when a GenAI-supported PC CDS tool serves as the first point of guidance in patient-facing use cases (eg, chronic health management and treatment planning). There may be usability challenges if digital and health literacy levels, community-specific factors, and local context are not considered during the design of GenAI-supported tools [60,61]. Additionally, chief medical information officers and other executives are essential since GenAI-supported tools require technological and financial resources to deploy and train care teams on their effective use. This creates the potential to exacerbate the digital divide between patients in health care organizations with robust health information technology support systems versus patients in lower-resourced health care organizations, leaving some patients without access to GenAI-supported self-management, communication, or monitoring tools that can help them recognize when care is needed and/or remain engaged between visits.

### Development of Clinical Decision Support Artifacts and Scaling

In addition to the clinician and patient and caregiver use cases, GenAI has the potential to improve the technology driving PC CDS. It can write code, map variables, create value sets [15], and generate realistic synthetic patient data that reduce the time needed for training and validation cycles [62]. GenAI can also suggest improvements to PC CDS logic [19] by analyzing alert overrides that contribute to clinician fatigue and personalizing alert criteria [63]. Furthermore, GenAI could solve a critical challenge in health care interoperability: the use of multiple incompatible information systems to record health data [64]. By using LLMs to quickly transform heterogeneous data into structured, standardized formats, it can facilitate the sharing of patient information across health systems. Despite this potential, developing interoperable GenAI systems is complex due to the quality of available patient data, the lack of implementation frameworks and standards for GenAI applications, and the cost of computational resources [65].

## Areas to Advance GenAI-Supported PC CDS

### Overview

The successful integration of GenAI in PC CDS across the use cases requires health system leadership, researchers, developers, informaticians, and policymakers to address at least the 6 areas discussed in subsequent sections to ensure these tools deliver meaningful, safe, and unbiased benefits. As with the benefits and challenges, these areas of opportunity are interconnected in nature and vary in relevance based on the clinical context, the decisions being made, and the patient-clinician interactions involved in the use case. Several of these areas align with the National Academy of Medicine’s AI Code of Conduct framework for the development and application of responsible AI in health and medicine [66]. In addition, since PC CDS does not occur in isolation, its success depends on effective public health strategies that promote community health and well-being.

### Engaging and Ensuring Representation of Patients and/or Caregivers in Design and Development

To capitalize on GenAI’s ability to support the PC CDS use cases illustrated in this manuscript, engaging patients and caregivers during the design and development process is essential [67]. As reflected in the National Academy of Medicine’s AI Code of Conduct, by involving end users in usability testing and other design activities—particularly those with varying language preferences, cultural norms, and digital and health literacy—developers can ensure GenAI-supported tools are tailored to account for the unique preferences, contexts, communication styles, and needs of individual patients [68,69]. Additionally, patient input can help ensure personalized treatment recommendations provided by GenAI are actionable for different populations [70] and accommodate the complexity of patients with multimorbidities. The degree to which patients and caregivers influence the design of GenAI-supported PC CDS tools will vary by use case, with greater influence typically possible in patient-facing applications and more limited involvement in clinician-facing functions such as diagnostic support or risk prediction.

### Building the Science of Effective PC CDS Implementation to Support Patient Engagement

Advances in methods to promote the adoption of evidence-based practices are needed to address the barriers to patient engagement posed by patient-facing GenAI-supported PC CDS in particular. Research is needed on the multilevel factors that (1) influence the use of communication support tools such as chatbots and digital health assistants, such as inaccurately processing patient inputs or lack of personal connection [70]; (2) support their seamless integration into clinical workflows and patient lifeflows so that the information provided by the PC CDS does not undermine clinician communication; and (3) support GenAI’s ability to facilitate SDM through visualizations that contain the appropriate level of detail [71,72]. A randomized clinical trial for a GenAI-enabled clinical decision aid used some of the aforementioned strategies through the incorporation of PRO measures, patient education, and tailoring to patient preferences, resulting in improvements in patients’ decisional quality, SDM, and functional outcomes [73]. Although the research on effective PC CDS implementation is nascent, this evidence supports the importance of further exploring multilevel factors influencing patient engagement to improve uptake of GenAI-supported PC CDS.

### Developing Risk-Based Policies for Deciding When GenAI Use Is Appropriate and What Level of Clinician Involvement Is Required

GenAI’s ability to integrate into such a wide range of PC CDS use cases highlights the importance of developing clear policies for its use. Risk-based guardrails should define when GenAI can operate autonomously in supporting patients’ knowledge and data capture (eg, by translating clinician-authored recommendations into plain language, generating visit summaries, or helping patients record symptoms and goals for minor issues) and where clinician review and sign-off are required before outputs are used to guide care (eg, for patients with multimorbidities who may face conflicting guidelines) [74]. Human-in-the-loop represents a promising approach for risk-based guardrails. Although its current use has largely focused on clinician-facing use cases such as verifying the accuracy and reliability of AI-generated discharge summaries or personalized recommendations, it also has potential to support patients with low digital health literacy in patient-facing use cases such as self-management support, communication support, and monitoring systems, where information is delivered digitally and patients may require additional support in interpretation. As seen in SDM research, patients with low digital health literacy benefit from clinician facilitation to interpret and apply complex health information, suggesting similar human-in-the-loop support may be beneficial for patient-facing GenAI-supported PC CDS tools [75]. While organizations bear primary responsibility for ensuring the safe and appropriate use of the GenAI tools they deploy, patients’ own GenAI literacy can also play a role in promoting appropriate use. Critical health AI literacy, the ability to critically evaluate GenAI outputs, recognizing how health systems and structural factors may shape them, and using this awareness to make informed decisions, is an emerging concept that can complement organizational safeguards by helping patients engage more safely and effectively with GenAI PC CDS [76]. Overall, well-defined policies will support postdeployment monitoring and help mitigate risks such as overreliance on GenAI, unclear liability, and inappropriate delegation of clinical judgment to technology.

### Establishing Independent Testing and Vetting Criteria to Ensure Safety and Accuracy

To ensure the quality and safety of GenAI-supported PC CDS, developers must rigorously evaluate and independently test processes before deployment [74]. Unlike static decision support systems, GenAI-supported tools require initial validation not only for accuracy but also for fairness, interpretability, and robustness against biases [46]. Suggested approaches include conducting centralized testing through third parties, as well as requiring local oversight by adapting models such as the Clinical Laboratory Improvement Amendments for AI [77]. Establishing standardized oversight for GenAI helps build trust in its recommendations and ensure its deployment improves, not compromises, care quality.

### Periodically Reassessing to Ensure No Algorithmic Drift and Verify Performance

GenAI algorithms and the data they operate on evolve, necessitating reassessment by the organization implementing GenAI-supported PC CDS to identify and address algorithmic drift, where shifts in data patterns lead to degraded performance or unintended outputs [74]. Because GenAI-supported PC CDS is integrated into real clinical workflows, monitoring approaches and metrics need to remain feasible and usable by clinical and organizational leaders, not just data scientists and researchers [78]. Several early prototypes, such as recent work developing human-centered AI monitoring systems for health care, demonstrate the utility of evaluating models along 4 core dimensions: performance, process, outcomes, and fairness [78]. For GenAI-supported PC CDS, outcomes and fairness can be assessed using similar principles, that is, evaluating whether recommendations lead to desired clinical and safety outcomes overall and across patient subgroups. Performance and process evaluation are more complex for GenAI because outputs are nondeterministic and language-based, and validated measures and methods are still emerging. Monitoring must therefore address behaviors such as hallucinations [79], reasoning quality [80], sycophantic behavior [81], and input robustness or feature drift [82]. While some elements require human expert review, automated and semiautomated tools are emerging. Examples include LLM-as-judge for evaluating correctness and guideline adherence or assessing fidelity to expected answers or text-based summaries of clinical information [83]; and statistical monitors that track token-level patterns or vocabulary shifts indicating changes in model behavior [82]. Organizations should establish clear triggers for reassessment—such as major guideline updates, new evidence affecting care recommendations, demographic changes in the patient population, the passage of time, or early signals of performance deterioration—and establish a reassessment frequency based on the clinical risk and operational impact of the GenAI-supported application [84,85].

### Establishing Policies to Promote Transparency and Patient Consent in the Use of GenAI

Patients need clear explanations of when and how GenAI is used in PC CDS tools that influence their care, along with the technology’s limitations [86]. The fair, appropriate, valid, effective, and safe principles [87] offer a blueprint for providing this transparency through specific, standardized technical and performance information. Furthermore, GenAI-supported PC CDS tools may require access to sensitive health information (eg, ambient listening tools and monitoring systems) with which patients may have varying levels of familiarity and comfort. Patients should have the right to opt out of the use of GenAI in their care and have full knowledge of who will access their data and how it will be used if they choose to participate. The development of patient-centered transparency and consent guidelines represents a critical area for advancing GenAI-supported PC CDS, including establishing which information is most relevant for patients to understand, determining how it should be effectively communicated, and considering when dynamic consent models may be warranted given the adaptive nature of GenAI systems [88].

### Conclusions

The integration of GenAI into PC CDS holds promise for transforming health care delivery. GenAI can enhance the personalization and precision of care; empower patients through improved engagement and SDM; and support clinicians, patients, and caregivers in making more informed decisions. However, the successful implementation of GenAI in PC CDS requires consideration of several critical factors. These include addressing potential biases in GenAI algorithms, ensuring the transparency and explainability of GenAI-driven recommendations, and fostering trust among patients and clinicians. Additionally, it is essential to engage patients and caregivers in the design and development of GenAI-supported tools, develop clear policies for GenAI use, and establish rigorous testing and validation processes to ensure safety and accuracy. By addressing these challenges, the full potential of GenAI can begin to be harnessed to improve patient outcomes and advance the field of PC CDS.

## Abbreviations

AI: artificial intelligence
EHR: electronic health record
GenAI: generative artificial intelligence
LLM: large language model
CDS: patient-centered clinical decision support
PGHD: patient-generated health data
PRO: patient-reported outcome
SDM: shared decision-making
